# The role of structural and process quality of family planning Care in Modern Contraceptive use in Indonesia: a multilevel analysis

**DOI:** 10.1186/s12889-021-11858-7

**Published:** 2021-10-05

**Authors:** Nurjaeni Nurjaeni, Yothin Sawangdee, Umaporn Pattaravanich, Charamporn Holumyong, Aphichat Chamratrithirong

**Affiliations:** Institute for Population and Social Research, Mahidol University, Nakhon Pathom, Thailand

**Keywords:** Contraceptive, Quality, Family planning, Indonesia, Multilevel, SDP, PMA2020

## Abstract

**Introduction:**

Despite contraceptive behaviors are influenced by multiple and multilevel variables, studies on modern contraceptive use in Indonesia has concentrated on single-level and mostly individual and household variables, and less interest has been devoted to multilevel analysis that accounts for community and SDP characteristics that may affect woman’s decision to use modern FP method. This study aimed to assess the role of structural and process quality of family planning care in modern contraceptive use among women in reproductive ages in Indonesia.

**Methods:**

This study analyzed data from the 2016 PMA2020 survey of 10,210 women in 372 enumeration areas in Indonesia. The data were analyzed using categorical principal component analysis and multilevel mixed-effects logistic regression.

**Results:**

The key variables for structural quality were number of contraceptive provided, SDP supports CHWs, available water and electricity, and skilled FP personnel, while the main factors for process quality were privacy of clients and provision of post-abortion service. There were significant differences across communities in how study variables associated with modern FP adoption. The finding shows the evidence of significant roles of structural and process quality FP care in modern contraceptive use. Moreover, women with high autonomy in FP decision, those who had free national/district health insurance, and those living in a community with higher proportion of women visited by CHW, had higher odds of modern contraceptive usage. Yet, women who live in a community with higher mean ideal number of children or greater proportion of women citing personal/husband/religion opposition to FP, had lower odds of modern contraceptive use than their counterparts.

**Conclusion:**

Study findings suggest improvement in structural and process quality of FP care will yield substantial growths in modern contraceptive use. Moreover, FP workers should also address adverse cultural/traditional customs in community and should target communities where the demand for modern FP was degraded by opposing social beliefs and norms. There was significant variation across communities in how individual, household, community, and SDP factors affect modern FP practice, hence, context should be taken into consideration in the development of FP intervention and promotion programs.

## Background

Indonesia experienced 8600 maternal deaths annually [1], and maternal mortality ratio (MMR) in Indonesia stays high as 305 deaths per 100,000 live births in 2015 [2, 3]. Indonesian MMR is comparatively the highest in Southeast Asia and is roughly 12 times higher than Thailand’s (25 deaths per 100,000 live births), 9 times compared to Malaysia, 5 times of Vietnamese, and around twice of Cambodian [1]. One of the imperative aspects in reducing maternal deaths is Family Planning. Scientific evidence had disclosed that modern contraceptive method use could decrease maternal deaths by about 35% [4, 5].

After the success story of Indonesian family planning (FP) program in enhancing maternal and child health, there are concerns about the failing to achieve the targets in increasing the utilization of modern contraceptive methods [2, 3, 6]. By December 2020, the Track20 had assessed the progress toward Family Planning 2020 initiatives which showed that Indonesia had achieved around 2.2 million additional contraceptive users, still far below the target of 2.8 million [7]. Moreover, for the past decade there was a stagnation in the attainment of the Family Planning program, the utilization of modern contraceptives in Indonesia is remain low at about 55% [8], contrasted to neighbor nations such as Vietnam and Thailand at 69% and 76% respectively [9–13]. Modern contraceptive use even fell from 58% in 2012 [14] to 57% in 2017 [6] and 55% in 2019 [8].

Furthermore, scientific evidences exposed that the share of unintended childbearing in Indonesia was almost one fifth, primarily caused by non-use of modern family planning methods, and a nearly 16% of unintended births could be prevented by modern contraceptive use [15, 16]. Studies by UNFPA Indonesia [17, 18] also disclosed a variety of multifaceted problems and concerns in access and quality of family planning care, such as high contraceptive stock out rates, deficiency and unequal distribution of trained health personnel including midwives, and so on.

Low modern contraceptive use and high unintended childbearing pose substantial encounters for Indonesia to achieve sustainable development goals. Despite contraceptive behaviors are influenced by multiple and multilevel variables, studies on modern contraceptive utilization in Indonesia had concentrated on single-level analysis and mostly individual and household variables, and less interest had been devoted to multilevel analysis that accounts for community and family planning provider characteristics that may affect woman’s decision to use modern contraceptive method. There is also a gap in the inconclusive role of quality family planning care and its structural and process elements in modern family planning methods’ utilization [19]. Assessing quality of family planning care and modern contraceptive use is very important for policy makers and program managers in designing and implementing quality-related interventions to enhance modern contraceptive use and subsequently improve maternal and child health in Indonesia.

While health professionals think that enhancing quality of care is essential, they have not reach a fixed consensus yet which elements should be contained within the definition of quality of care [20–26]. In basic concept, quality means excellence, expected outcome that is aimed to be achieved [27, 28]. Traditionally, quality of care has been termed as providing practically skilled, effectual, risk-free health services which support to the client’s well-being [29–34]. World Health Organization (WHO) defined quality of care as “the degree to which delivered health care services enhance preferred health outcomes” [35, 36]. However quality of care is a multidimensional subject which could be defined and measured in a various way [20–26]. As a consequence, recommendations have been proposed to develop the range of quality of family planning care to involve various elements of health care [37]. While several quality care frameworks have substantiated numerous research on the quality of family planning care, no single framework has been considered as gold standard [20–26]. Hence, these manifold standpoints have permitted scholars to use different framework and distinct aspects of quality of care that most fit their research objectives [20–22, 38–40]. One of the earliest, standard, and world widely used framework that define the quality of care was constructed by Avedis Donabedian [41] who study extensively on the quality of care. Donabedian [41] defined quality of care as the implementation of health science and medical technology in an approach that maximizes the advantages without increasing the health risk and considered quality of care regarding 3 key factors:

1. Structural quality such as infrastructure, equipment, human and capital resources, management, availability of contraceptive choices, days of services open for clients, etc.;
2. Process quality such as interpersonal relations, counselling, client’s privacy/confidentiality, etc.
3. Outcome quality such as user’s satisfaction, shifts in Family Planning behaviors, etc.

This study, hence, followed Donabedian framework and was focused on two elements of quality FP care, which were structural quality and process quality of family planning care.

The aim of this analysis was to assess correlates of modern contraceptive use in Indonesia, especially the role of structural and process quality of family planning care in modern contraceptive use among women in reproductive ages in Indonesia. It is expected that better structural and process quality of family planning care given by the closest contraceptive provider would increase the likelihood of women who lived nearby to use modern family planning methods. Two summary index of quality FP care were developed for each service delivery point (SDP) to investigate this relationship: one for structural quality index, and another one for process quality index. Each women respondents then linked to their closest SDP by Global Positioning System (GPS) points. The role of quality family planning care of the closest SDP in woman’s modern contraceptive use was then analyzed using two-level regression model to deal with cluster-effect within the data.

## Data and methods

### Data source

This research analyzed data from the round 2 (last round) of Performance Monitoring and Accountability 2020 Indonesia (PMA2020 Indonesia) survey that collected nationally representative data from women in reproductive ages and SDP about family planning which also collected data on quality of family planning care [42]. Two PMA2020 datasets of women in reproductive ages and SDP were gathered at the same period and can be linked by Global Positioning System (GPS) points of household and SDP. This research was thus allowed to examine the role of closest SDP quality contraceptive care in women’s modern contraceptive behavior. Data were extracted from the 2016 PMA2020 Indonesia survey. A multi-stage stratified cluster sampling design was employed, with 372 enumeration areas (EAs) was randomly chosen from all 34 provinces of Indonesia based on a national sampling frame yielded from the latest population census before the survey. In this study, one enumeration area was regarded as one community with its size varied from 200 to 500 households, and then thirty-five households were randomly chosen from each community/enumeration area and all the household members enumerated. Afterwards, all females in childbearing ages in the households were given informed consent and were then interviewed. From each enumeration area, one to three private health facilities were also randomly selected and subsequently interviewed. Furthermore, three types of public health facilities serving the population in each enumeration area were surveyed as well, which were the public health post, primary health center, and public hospital. All of these public and private health facilities hence was representative of the health service provision accessible to the women population in the enumeration area.

Both household and SDP datasets were merged based on the EA-ID variable within the two datasets. The samples in these analysis were all household members who were women in ﻿childbearing age (15-49 years) that slept in the household the night before the interview and only SDPs offering family planning services to the women population in their respective enumeration area (EA). Data of 992 SDPs were successfully merged to the household dataset of 10,210 women in reproductive ages (15–49 years) in 372 communities.

### Dependent variable

The dependent variable of this analysis was current use of modern contraceptive methods, categorized as ‘Yes’ for those who use any modern contraceptive method, and ‘No’ for those who utilize traditional contraceptive method and those who do not use any modern or traditional method at the time of survey.

### Individual and household (level 1) variables

The individual (level 1) factors consist of ﻿female’s age (15-19 years, 20-24 years, 25-29 years, 30-34 years, 35-39 years, 40-44 years, 45-49 years), educational attainment (none/primary school, junior high school, senior high school, or academy/university), parity (1, 2, 3, or more), marital status (never married, married/living with partner, or divorced/separated/widow), woman’s autonomy in FP decision (low or high), and health insurance (no insurance, free national/district insurance, non-free national insurance, or other insurance). In addition, household (level 1) factor include household wealth (poorest, poor, middle, rich, or richest).

### Community and SDP (level 2) variables

#### Community residence

The community residence factors comprise as follows:

1. Place of residence: rural or urban.
2. Region of residence: Sumatera (provinces of Aceh, North Sumatera, West Sumatera, Riau, Riau Islands, Bengkulu, Jambi, South Sumatera, Bangka Belitung, and Lampung), Java and Bali (provinces of West Java, Banten, Jakarta, Jogjakarta, Central Java, East Java, and Bali), Nusa Tenggara (provinces of West Nusa Tenggara and East Nusa Tenggara), Kalimantan (provinces of West Kalimantan, Central Kalimantan, North Kalimantan, South Kalimantan, and East Kalimantan), Sulawesi (provinces of North Sulawesi, Gorontalo, South Sulawesi, West Sulawesi, and Central Sulawesi), and Maluku and Papua (provinces of Maluku, North Maluku, Papua, and West Papua).

#### Community cultural/traditional norms

To estimate social/cultural beliefs and customs in community, we arranged two variables as follows:

1. The mean ideal number of children of women residing in the community (EA).
2. The proportion of women in community (EA) who cited dissatisfaction with family planning. A female respondent was categorized as being dissatisfied with family planning if she stated the reason of not using any FP method was due to personal, or husband, or religious opposition, or fear of side effects or other health concerns.

These two variables were constructed as community-level variables that was calculated within the cluster or enumeration area (EA).

#### Community exposure to demand generation efforts

To measure community exposure to demand-generating efforts, we calculated one community-level variable, which was the proportion of women in community (EA) who were visited by a community health worker who talked about family planning in the last twelve months.

#### Structural quality of family planning care

In this study, ten structural factors were summarized into one single index as ‘structural quality of family planning care’:

1. Available skilled health personnel: whether skilled health personnel were available on the day when SDP offers FP service.
2. Days serving Family Planning: number of days per week SDP offered family planning service.
3. Electricity & Water: whether SDP personnel had available access to electricity and water.
4. Fees charged for Family Planning: whether clients were charged for obtaining modern contraceptive method from the SDP.
5. Choice of method: number of modern contraceptive methods SDP offered to clients.
6. Contraceptive stock-out: whether SDP had no contraceptive stock-out in the last 3 months.
7. FP equipment: number of family planning equipment available in the examination room.
8. Examination room’s condition: whether examination room was clean, and storage of Family planning methods were protected from sun, water, and pests.
9. SDP visited by supervisor: whether SDP had been visited by a supervisor in the last 6 months.
10. SDP supports CHWs: whether SDP provided family planning supplies to community health workers (CHWs).

#### Process quality of family planning care

Four process factors were summarized into an index as ‘process quality of family planning care’:

1. Clients’ confidentiality: whether SDP maintained visual & auditory privacy in examination room.
2. Review of Clients’ Opinion: whether SDP collected and reviewed clients’ opinion.
3. FP care for adolescent: whether SDP counsels, or prescribes, or provides FP to adolescents.
4. Post-abortion service: whether SDP offered post-abortion service.

Other SDP-level variable included in the analysis was SDP type (hospital, health center/clinic, village health post/delivery post, private doctor/midwife, or others).

### Data analysis

The variables of main interest were the structural quality and the process quality of family planning care. The structural quality index was obtained from categorical PCA (principal component analysis) by synthesizing ten structural quality components of family planning care. Then, this structural quality index was categorized into three categories (low, medium, high) using the tertile value as cutoff reference as it divided the SDP data into three equal proportion of structural quality.

On the other hand, process quality index was also yielded from categorical principal component analysis by merged four variables related to process quality elements of Family Planning care. This process quality index was classified into three categories (low, medium, high) using the tertile value as cutoff reference as it divided the SDP data into three equal proportion of process quality.

Then authors matched each individual respondent to their nearest SDP offering contraceptive services. The GPS coordinates of the SDP and female’s household were used to calculate the closest distance to choose the nearest facility for each individual female respondent.

Multilevel regression model was performed to estimate female’s odds ratio (OR) of using a modern contraceptive method. Authors performed these model building approaches:

- First phase: A ‘null model’ without any independent variables was analysed to verify whether there was a significant variation across clusters/EAs regarding the odds of modern method usage. This empty model confirmed whether there was any significant random effect on multilevel model, by cheking whether the variance of multilevel model intercept was significant or not.
- Second phase: A full or comprehensive model with all explanatory variables (level 1 and level 2) was then built to assess the independent influence of structural and process quality of family planning care on modern contraceptive use after adjusted for all other covariates.

Variance Inflation Factors (VIF) scores was calculated to identify whether there was multicollinearity in the regression analysis or not. *P*-values with α equal to 0.01, 0.05, and 0.1 were used in the model. Included samples in the analysis were weighted and the variances of the explanatory factors were adjusted accordingly. We used SPSS 20 for Categorical PCA and Stata 14 for the rest of analyses.

## Results

### Summary index of structural quality of family planning care

**Fig. 1:**
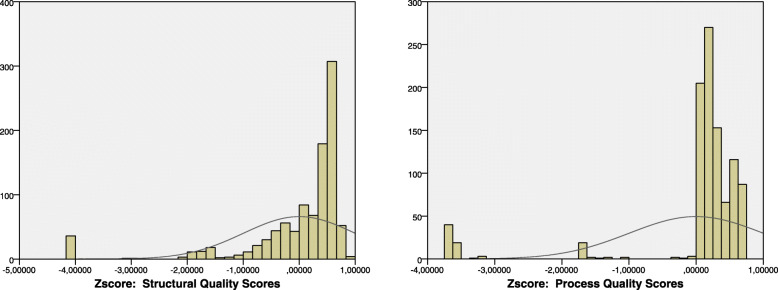
Normalized Scores for Structural Quality Index and Process Quality Index

The results of descriptive and Categorical PCA analysis of structural quality FP care of the nearest SDPs are displayed in Table 1. The categorical PCA analysis had identified the first component explaining main variation across all SDPs for the 10 structural indicators (Table 1). The first component had eigenvalue of 3.674 and Cronbach’s alpha values of 0.809. Number of modern contraceptives provided by SDP and support for CHWs were the greatest loading factors, which means it is the key variable in structural quality that described the major variation accross 992 SDPs (0.887 and 0.886 respectively). Protected contraceptives and clean examination room (0.655), available trained health personnel (0.771), and available water and electricity (0.855) were also highly loaded. On the other hand, free of charge family planning service and number of days SDP offering family planning service both had a low loading (− 0.261 and − 0.159 respectively). The normalized structural quality scores are displayed in Fig. 1.

**Table 1:** Categorical Principal Component Analysis for the structural quality and process quality of Family Planning care of nearest SDP, PMA2020 Indonesia survey, 2016 (*n* = 992)

Indicators	n	%	mean	SD	Factor Loadings
***Structural Factors:***					
1. Number of days per week SDP offering FP			5.85	1.29	−0.159
2. Free of charge FP service	304	30.65			−0.261
3. Available skilled FP personnel	715	72.08			0.771
4. Number of modern methods provided			4.96	1.63	0.887
5. No stock out in last 3 months	409	54.75			0.316
6. Number of equipment in examination room			9.53	2.17	0.329
7. Available water and electricity	724	75.97			0.855
8. Examination room is clean and contraception were protected	745	75.11			0.655
9. SDP was supervised in last 6 months	887	90.79			0.213
10. SDP supports Community Health Workers	376	40.47			0.886
Eigenvalue					3.674
Cronbach’s alpha					0.809
***Process Factors:***					
1. Privacy and confidentiality of client in examination room	405	44.85			0.945
2. Client’s opinions is collected & reviewed by SDP	278	28.02			0.299
3. SDP counsel, or prescribe, or provide FP for adolescent	544	55.23			−0.096
4. SDP provide post abortion service	578	62.22			0.944
Eigenvalue					1.882
Cronbach’s alpha					0.625

### Summary index of process quality of family planning care

The categorical PCA analysis (see Table 1) had also yielded the first dimension/component describing major variation among SDPs for the 4 process factors (Table 1). The first dimension had eigenvalue of 1.882 and Cronbach’s alpha values of 0.625. Confidentiality of clients (both visual and auditory privacy) and provided post-abortion service were the highest loading indicators that means both are the key variables in process quality that explained the main variation accross all the SDPs (0.945 and 0.944 respectively). On the other hand, collection and review of clients’ opinion (0.299) and family planning service for adolescent (− 0.096) both had a low loading. The normalized process quality scores are showed in Fig. 1.

### Study Population characteristics

**Fig. 2:**
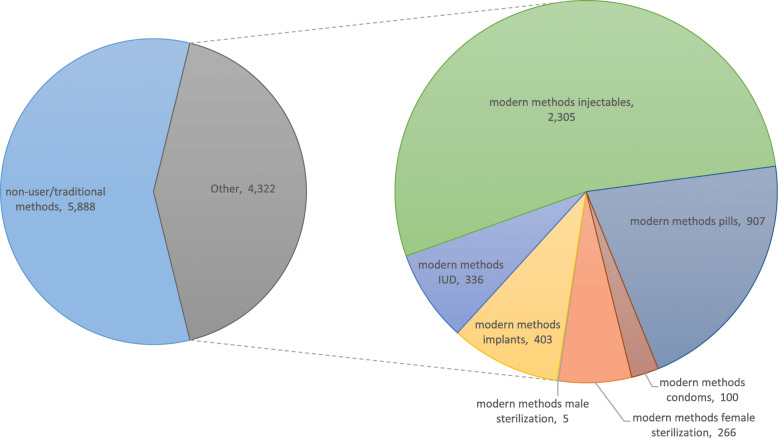
Current use of family planning method among women in reproductive ages, PMA2020 Indonesia, 2016 (*n* = 10,210)

As we can see in Figs. 2, 10,210 women in reproductive ages from 372 communities (EAs) were analyzed in this study. In the 2016 PMA2020 Indonesia survey, 42.33% of women in childbearing ages stated that they were using a modern contraceptive method and 57.67% were either non-users or using a traditional family planning method. Among 4322 modern contraceptive users, about 53.33% were using injectables, 20.99% were using pills, 9.32% were implant users, 7.77% were IUD users, 6.15% were using female sterilization, 2.31% were using condoms, and around 0.12% women stated that their husband/partner were using male sterilization.

Individual and household characteristics of study population are portrayed in Table 2. Among women in childbearing ages, majority of women were aged 35–39 (16.44%), had secondary/high school educational attainment (57.32%), were married or living with a partner (71.87%), had no child (28.39%) or had two children (26.75%), had a higher autonomy in decision for using or not using FP (59.01%), had a high wealth quintile (25.38%), but had no health insurance (39.22%).

**Table 2:** Individual and Household characteristics of women in PMA2020 Indonesia, 2016 (*n* = 10,210)

Variable	Frequency (N)	Percentage (%)
Age		
15–19	1477	14.47
20–24	1303	12.76
25–29	1292	12.65
30–34	1532	15.00
35–39	1679	16.44
40–44	1586	15.53
45–49	1341	13.13
Women education level		
No education / Primary School	2868	28.09
Secondary (High School)	5852	57.32
Higher (Academy/University)	1490	14.59
Parity ^**a**^		
No child	2896	28.39
1 child	1823	17.87
2 children	2728	26.75
3 children	1581	15.50
More than 3 children	1171	11.48
Marital status		
Never married	2433	23.83
Married/Living with partner	7338	71.87
Divorced/Separated/Widow	439	4.30
FP Decision Autonomy (Use FP/Not use FP)^**b**^		
Low (Decided by other/s)	4061	40.99
High (Decided by oneself)	5846	59.01
Wealth index		
Poorest	1675	16.41
Poor	1791	17.54
Middle	1873	18.34
Rich	2591	25.38
Richest	2280	22.33
Health insurance		
No health insurance	4004	39.22
Free National/District Health Insurance	2165	21.20
Non-free National Health Insurance	1975	19.34
Other health insurance	2066	20.24

^**a**^ 11 missing values

^**b**^ 303 missing values

Community characteristics of study population are figured in Table 3. Among 10,210 women under this study, majority of women lived in urban area (55.22%) and reside in Java and Bali region (48.72%). On an average, respondents lived in a community where mean ideal number of children were 2.66 children with a standard deviation of 0.48 children, 15.05% women in community had cited a dissatisfaction with Family Planning, and 9.49% of women had been visited by a Community Health Worker who talked about Family Planning in the last 12 months.

**Table 3:** Community and nearest-SDP characteristics of women in PMA2020 Indonesia, 2016 (n = 10,210)

Variable	Frequency(N)	Percentage (%)
Place of residence		
Rural	4572	44.78
Urban	5638	55.22
Region of residence		
Sumatera	1870	18.32
Java and Bali	4974	48.72
Nusa Tenggara	386	3.78
Kalimantan	489	4.79
Sulawesi	2271	22.24
Maluku and Papua	220	2.15
Community-level Mean Ideal Number of Children	2.66 (mean)	0.48 (SD)
Percent of women cited a dissatisfaction with FP	15.05 (mean)	12.52 (SD)
Percent of women had been visited by CHW about FP in last 1 year	9.49 (mean)	12.59 (SD)
SDP type		
Hospital	249	2.44
Health center/clinic	5924	58.02
Village Health Post/Delivery Post	1923	18.83
Private Doctor/Midwife	1539	15.07
Others	575	5.63
Structural Quality of FP Care ^**c**^		
Low	2185	22.73
Medium	4063	42.27
High	3364	35.00
Process Quality of FP Care ^**c**^		
Low	2771	28.83
Medium	4175	43.44
High	2666	27.74

^**c**^ 598 missing values

### Random effect and multilevel model diagnostic

Multilevel mixed-effect regression analysis was conducted to determine the roles of individual and household (level 1) variables and community and SDP (level 2) variables in current use of modern contraception. The findings are displayed in Table 4 and Table 5. The null model indicates substantial inequalities in modern contraceptive usage across communities (EAs) with the community-level variance of 0.49 and a median odds ratio of 1.95 (see Table 4). It is indicated that there was significant clustering effects and all of the observations in the data cannot be treated as independent using single level model. The multilevel mixed effects models are a valid and adequate approach to analyzing this data.

**Table 4:** Measure of model fitness

Characteristics	Indicators	Null Model	Multilevel Model
Groups	Number of communities (EAs)	372	372
PSU	Number of women	10,210	10,210
Random effect	Variance (constant)	0.490	0.114
	95% CI	0.396–0.605 ^**^	0.066–0.195 ^**^
	Median Odds Ratio	1.9498	1.3799
	95% CrI	1.846–2.054 ^**^	1.319–1.441 ^**^
Model diagnostics	Log-likelihood	− 6703	−3829 ^**d**^
	AIC	13,411	7740 ^**d**^
	BIC	13,425	8032 ^**d**^
Multicollinearity	Mean VIF	–	1.33

PSU = Primary Sampling Unit; CI = Confidence Interval; CrI = Credible Interval;

AIC = Akaike information criterion; BIC = Bayesian information criterion

^**^ Significant at *p* < 0.05; ^**d**^ Fitted statistics

VIF = Variance Inflation Factors

**Table 5:** Adjusted odds ratio ^**f**^ from multilevel mixed-effect regression of modern contraceptive utilization among women in reproductive ages (15–49 years), PMA2020 Indonesia survey, 2016

Characteristics	AOR ^f^	(95% CI)
Age		
15–19	1	
20–24	0.94	(0.55–1.61)
25–29	0.47	(0.27–0.80)***
30–34	0.32	(0.19–0.54)***
35–39	0.27	(0.16–0.47)***
40–44	0.19	(0.11–0.33)***
45–49	0.09	(0.05–0.15)***
Women education level		
No education / Primary School	1	
Secondary (High School)	1.14	(0.99–1.30)*
Higher (Academy/University)	0.96	(0.78–1.20)
Parity		
No child	1	
1 child	29.51	(17.63–49.38)***
2 children	77.99	(46.06–132.03)***
3 children	111.49	(65.08–190.99)***
More than 3 children	96.90	(55.92–167.91)***
Marital status		
Never married	1	
Married/Living with partner	50.34	(19.36–130.87)***
Divorced/Separated/Widow	1.11	(0.38–3.23)
FP Decision Autonomy (Use FP/Not use FP)		
Low (Decided by other/s)	1	
High (Decided by oneself)	2.81	(2.48–3.18)***
Wealth index		
Poorest	1	
Poor	1.06	(0.87–1.30)
Middle	0.95	(0.78–1.17)
Rich	0.99	(0.81–1.22)
Richest	1.00	(0.80–1.26)
Health insurance		
No health insurance	1	
Free National/District Health Insurance	1.14	(0.98–1.33)*
Non-free National Health Insurance	1.10	(0.93–1.30)
Other health insurance	1.12	(0.95–1.31)
Place of residence		
Rural	1	
Urban	0.74	(0.63–0.86)***
Region of residence		
Sumatera	1	
Java and Bali	1.30	(1.07–1.58)***
Nusa Tenggara	0.65	(0.45–0.95)**
Kalimantan	1.48	(1.04–2.11)**
Sulawesi	0.67	(0.54–0.85)***
Maluku and Papua	1.09	(0.63–1.86)
Community - Mean Ideal Number of Children	0.55	(0.47–0.64)***
Percent of women cited a dissatisfaction with FP	0.96	(0.96–0.97)***
Percent of women had been visited by CHW about FP in last 1 year	1.01	(1.00–1.01)**
SDP type		
Hospital	1	
Health center/clinic	0.99	(0.61–1.61)
Village Health Post/Delivery Post	1.21	(0.73–1.99)
Private Doctor/Midwife	1.09	(0.66–1.81)
Others	1.41	(0.81–2.47)
Structural Quality of FP Care		
Low	1	
Medium	1.00	(0.83–1.21)
High	1.28	(1.05–1.55)**
Process Quality of FP Care		
Low	1	
Medium	1.19	(1.01–1.41)**
High	1.19	(0.99–1.42)*

^**f**^ Adjusted for all other covariates included in the study

AOR: adjusted odds ratio; 95% CI: Confidence Interval; 1: Reference category

* *P* < 0.10; **P < 0.05; ***P < 0.01

In relation to model fitness, the multilevel model which included all the individual, household, community and SDP level variables had lower AIC of 7740 and lower log-likelihood ratio of − 3829, therefore was believed as fitter model for estimating the event of modern contraceptive utilization among women in childbearing ages than the null model (Table 4). The likelihood ratio test also shows that the multilevel model was significantly better than the single-level model and the null model (result is not shown). The mean of Variance Inflation Factors (VIF) for all covariates were 1.33 (less than 10) indicates no multicollinearity within the multilevel model.

### Correlates of modern contraceptive use

#### Individual and household level factors

The two-level mixed-effect logistic model (Table 5) disclosed that the odds of using modern contraceptives decrease along woman’s age after adjusted for all other covariates (Table 5). Women with age between 25 and 29 (AOR = 0.47; 95% CI: 0.27–0.80), 30–34 (AOR = 0.32; 95% CI: 0.19–0.54), 35–39 (AOR = 0.27; 95% CI: 0.16–0.47), 40–44 (AOR = 0.19; 95% CI: 0.11–0.33), and 45–49 (AOR = 0.09; 95% CI: 0.05–0.15) were less likely to use modern contraceptives compared to women in the youngest age group. Table 5 also discovers that when adjusting for other factors, the odds of modern contraceptive utilization raised along the parity a woman had. Those having one child or two children had an approximated 29.51 (95% CI: 17.63–49.38) and 77.99 (95% CI: 46.06–132.03) times higher odds of modern contraceptive usage, respectively, than women having no child; and those having three children or more than three children had 111.49 (95% CI: 65.08–190.99) and 96.90 (95% CI: 55.92–167.91) times higher odds of modern contraceptive use than women with no parity. The model also estimates that respondents who had attended high school had 1.14 (95% CI: 0.99–1.30) times higher odds of modern contraceptive utilization than those having no education or primary school (Table 5). The odds of using modern contraception was 2.81 (95% CI: 2.48–3.18) times higher among women who had high autonomy in FP decision than among women who had low autonomy. Free national/district health insurance also statistically rose the odds of modern contraceptive use by a factor of 1.14 (95% CI: 0.98, 1.33). Yet, household wealth had no substantial role in the use of modern contraception; those with higher household wealth had no significant difference in the likelihood of using modern contraceptive than those with lower household wealth.

#### Community and SDP level factors

After adjusting for other indicators, females who reside in urban area had 26% (AOR = 0.74; 95% CI: 0.63–0.86) lower odds of modern contraceptive use than those reside in rural area (Table 5). Women in Java-Bali and Kalimantan had 1.3 time (AOR = 1.30; 95% CI: 1.07–1.58) and 1.48 time (AOR = 1.48; 95% CI: 1.04–2.11) higher likelihood to utilize modern contraception, respectively, compared to those who reside in Sumatera. However, women in Nusa Tenggara and Sulawesi were 35% (AOR = 0.65; 95% CI: 0.45–0.95) and 33% (AOR = 0.67; 95% CI: 0.54–0.85) less likely for using modern contraception, respectively, contrasted to those residing in Sumatera (Table 5). Furthermore, as the proportion of women who dissatisfied with FP in a community increased, the likelihood of modern contraceptive utilization for each woman in the community significantly decreased (AOR = 0.96; 95% CI: 0.96–0.97). Likewise, the more common it was for community members to have higher ideal number of children, the least likely it was for each woman residing in the community to use modern contraceptive (AOR = 0.55; 95% CI: 0.47, 0.64). Oppositely, it was evident that as the percentage of community members had been visited by CHW concerning FP increased, so did the likelihood of modern contraceptive usage for each woman in the community (AOR = 1.01; 95% CI: 1.00–1.01). Moreover, the likelihood of modern contraceptive use were 1.19 (AOR = 1.19; 95% CI: 1.01–1.41) times significantly greater among women who live near a high process-quality or medium process-quality SDP contrasted to those who live near a low process-quality SDP. In addition, the likelihood of modern contraceptive usage were even 1.28 times higher for women residing near a high structural-quality SDP than a low structural-quality SDP (AOR = 1.28, 95% CI: 1.05–1.55). However, all closest SDPs regardless of their type shows no significant difference in modern contraceptive adoption.

## Discussion

This analysis had built summary measures for structural and process quality of family planning care by summarizing related indicators identified in the previous studies into two composite index by employing categorical Principal Component Analysis (CATPCA). The first component resulted from CATPCA for structural factors had a quite high Cronbach’s alpha and eigenvalue (0.809 and 3.674 respectively). Moreover, the first component yielded from CATPCA for process factors had a Cronbach’s alpha of 0.625 and an eigenvalue of 1.882. Both eigenvalues were higher than 1 and Cronbach’s alpha also indicated an adequate internal consistency, thus, this study was allowed to use the first component of both results as the summarize measures for structural and process quality FP care respectively.

After both quality scores were normalized, each of them were classified into three categories of low, medium, and high quality using the tertile values as cut-off reference. Both composite index were then included in a multilevel model analysis by linking every individual women to their nearest SDP to examine the role of structural and process quality of FP care in modern contraceptive use in Indonesia.

Furthermore, indicators that had high loading in the first component of structural quality index yielded from CATPCA analysis were SDP supports for Community Health Workers, contraceptives protected from sun, water, and pests plus clean examination room, available skilled health personnel, available water and electricity, and the highest loading was for number of contraceptives offered. Previous research had highlighted the broader range of contraceptives offered as one of the key factors of quality FP care indicating that SDP providing more contraceptives will produce better quality FP care [43–45]. In addition, other research had also found that method mix provided by SDP plays a crucial role in quality FP care [46–48]. The number of contraceptives offered was identified in this current analysis to be the highest loading, evidently stressing the significance of SDP having broader range of contraceptives, primarily as this essential for maintaining sustained use of modern contraceptives.

On the other hand, in the first component resulted from CATPCA analysis for process quality of FP care, provision of post-abortion service had a high loading while confidentiality of clients produced the highest loading. This finding suggests that visual and auditory privacy in the FP examination room are also imperative for quality FP care. The importance of maintaining clients’ confidentiality and privacy had been also underlined by prior studies as one of crucial factors for quality FP care [19, 49].

Our study reveals that modern FP uptake declined along woman’s age, adjusted for other correlates including number of living children. We found that modern contraceptive utilization was higher among younger women than older women, which agrees prior studies [50–52]. Previous research explained the higher usage of family planning among younger females is contributed by effectual inter-spousal communication on modern contraceptive substances [50–52]. We further identified a substantial relationship between marital status and modern contraceptive utilization, with married women and those living with partner were more likely to utilize modern FP method than women who were never married. Similar findings had been found in other research [53, 54]. Also, the odds of modern contraceptive usage raised along woman’s parity. The uptake of modern contraceptives was considerably greater among women with higher parity contrasted with those who were yet to have a child, this was similar to prior studies [52–56]. However, there was no substantial relationship found between modern FP uptake and household wealth, contrary to most common findings in prior studies [52, 56–59].

We also saw that educational attainment of woman affects modern contraceptive usage, with better uptake among those who attended high school compared to women with no education or primary school, this was in line with other research [52, 53, 56–59]. Consistent with prior studies [52, 59–63], we observed higher usage of modern FP method among women with higher autonomy in FP decision as contrasted with those who had lower autonomy in FP decision. This finding suggests that some community-based programs to enhance decision making power of women by increasing women’s awareness and their access to sexual and reproductive health information and services including family planning care would help to increase modern contraceptive use. Moreover, the use of modern FP methods was observed to be higher among women who had a free national/district health insurance. This finding confirms another evidence from prior study [64].

Our study exposes that rural women had better odds of using modern FP method than urban women. Yet, this was in line with PMA2020 Indonesia round 2 report [65]. It was exciting to acknowledge that women living in rural areas had a higher likelihood in modern contraceptive use. It may be rationalized by the national FP programs in Indonesia targeting rural women [66]. Therefore, it can be viewed as an achievement of national government intervention initiatives implemented at rural areas. We also found disparities in modern contraceptive usage across regions of Indonesia. Differences in modern contraceptive adoption across region in Indonesia had been identified as far back as the beginning of 1990’s [67]. These disparities have persisted for decades despite continuous governmental interventions and funding targeted for diminishing the inequalities in modern contraceptive use across region/province in Indonesia. Prior analysis cited that an unequal resources such as health infrastructures, contraceptive choices and human resources across regions as main factors for causing these inequalities across regions in Indonesia [2, 17, 18].

Our findings indicate that prevailing social/traditional customs a community had were statistically related to woman’s odds for using modern method after other covariates was taken into account. Particularly, staying in a community with higher proportion of women dissatisfied with FP had a negative influence on modern FP usage. The results also reveal that higher mean ideal number of children in a community was strongly related to lower modern FP use. This might occur because of the demand for modern FP seems to be deteriorated by prevailing cultural/traditional beliefs and norms including high ideal number of children and high level of opposition to FP that lead to dissatisfaction with FP [68–71]. Improving quality FP care and promotions programs, and expanding information, education, communication (IEC) in such communities would help to escalate community approval on modern FP use.

The findings further disclosed that proportion of community members had been visited by community health worker was positively associated with modern contraceptive utilization. This could be contributed by information, education, and communication (IEC) delivered by community health volunteer that may influence woman’s decision making process to use modern FP method [72–74]. This finding suggests community outreach / social mobilizer programs as an essential strategy to enhance modern contraceptive prevalence. Prominent increases in modern contraceptive utilization could be obtained by providing more supports for community health volunteers to address traditional beliefs and norms and to educate women in their community regarding healthy timing and spacing childbirths.

The findings of current study also identify significant roles of quality of family planning care in modern contraceptive use in Indonesia, this corroborates previous research on quality FP care [75–81]. After adjustment for other covariates, process quality of FP care had a statistically positive relationship to a woman’s likelihood for using modern FP method. The multilevel model also found an evidence of the significance of structural quality FP care for modern contraceptive use. The empiric role of process quality index recommends that the process quality of family planning care are vital to enhance modern contraceptive use. Respectively, the significant role of structural quality index also confirms that upgrading structural elements of quality family planning care could boost the utilization of modern contraceptive as well. This is essential due to limited scientific proof of direct associations between structural quality of FP care and modern contraceptive use. The existing significance of quality FP care in modern contraceptive use is mostly found on process elements of FP care [82]. The significant role of structural quality of FP care in modern contraceptive adoption has not been well known.

Jointly, current study findings emphasize that government and stakeholders should implement a multilevel approach in order to expand modern FP use, such as supporting enhancement in structural and process quality of family planning care and lifting traditional/cultural norms to generate a supporting atmosphere for demand generation of modern FP methods. Considering contextual barriers and facilitators, yet, is imperative for mounting up FP programs to achieve state and global reproductive health goals. For instance, despite continuous FP program strategic implementation in place, providing quality FP care is still an issue in many places. There are still many SDPs in Indonesia that do not have skilled health personnel such as doctor or midwife and more than one third of SDPs in Indonesia experiencing modern contraceptive stock out due to unequal distribution of skilled health personnel, inadequate supply chain management, and limited resources [2, 17, 18], that may lead to SDP lower quality FP care. Addressing these issue will need significant enhancements in FP program management and extra FP resources, including family planning infrastructures, skilled health personnel, and broader range of contraceptives. Besides, Community Health Workers may need more additional supports in educating their community members to diminish cultural/traditional norms that structurally opposing FP.

Overall, the findings indicate there was a low utilization of modern contraceptive methods among women of reproductive age in Indonesia. Hence, new FP strategic programs should be developed to raise modern FP practice among women in childbearing ages. Key facilitators and barriers of modern contraceptive use include not only individual and household characteristics, but also community and SDP level factors. Therefore, governmental and private interventions to escalate the use of modern FP methods in Indonesia should incorporate not only individual and household factors, but also community and SDP factors into consideration.

## Strength and limitations

Based on our literature review, no prior research had assessed the role of structural and process quality of family planning care in modern contraceptive use among women in reproductive ages, using multilevel analysis and composite index of structural and process quality FP care, to extract individual, household, community, and SDP level influences on modern contraceptive behavior in Indonesia. Another strong point was that this analysis employed the latest nationally representative survey data of women in reproductive ages and SDPs collected in the same period using international standardized questionnaires and geographic information system (GIS). This study has also covered a wider range of correlates of modern contraceptive practice than have been assessed in prior research in Indonesia, and has delivered some evidence of the significance of multiple and multilevel factors in modern contraceptive adoption in Indonesia, therefore, it has several applied propositions for FP programs in Indonesia.

Some shortcomings of this study are to be noticed, few are general weaknesses of all research deriving statistical inferences from variables’ associations using cross-sectional data, and one limitation are exclusive to this analysis. Firstly, interpretations regarding variables’ effect on the odds of modern contraceptive uptake are restricted by the characteristics of cross-sectional data used in the study. Future research can address this limitation by using panel or longitudinal study design for validating causal impact of structural and process quality FP care on modern FP uptake. Secondly, linking women to their nearest SDP supports this research’s capacity to estimate the role of structural and process quality FP care on modern FP usage; however, it needs assumption that nearest FP care provision affects woman’s FP attitude. Thirdly, this study has additionally covered structural elements of quality FP care that not included in Bruce framework that focused only on process quality factors, yet, this study has not covered client satisfaction factors included in Donabedian framework. Future studies can elaborate other important elements in quality FP care that might also have key role in modern FP use.

## Conclusion

Structural and process quality of family planning care were evident to have significant roles in modern contraceptive use in Indonesia. Higher structural quality and higher process quality were associated with higher modern FP method use. These findings suggest improvement in both structural and process quality of FP care will yield substantial growths in modern contraceptive utilization. Moreover, community mean ideal number of children, community-members proportion having dissatisfaction with FP, and community-members proportion had been visited by CHW were also identified as statistically significant factors for modern contraceptive uptake in Indonesia. These findings suggest that FP workers should also address prevailing traditional/social norms and should target communities where the demand for FP was degraded by cultural/social beliefs and customs. Viable FP intervention and promotion programs such as community outreach/mobilization with available, accessible, acceptable, and good quality FP care should also be arranged for such communities. Significant individual, social, and quality FP care factors in modern contraceptive use has been identified which FP program planners and managers can use. Thus, applying adequate works on these significant factors is substantively crucial as it has noteworthy public health advantages. However, it should be kept in mind that findings from multilevel mixed-effect regression analysis has demonstrated that there was significant variation across communities (EAs) in how individual, household, community, and SDP factors affect modern FP method use. Hence, context should be taken into consideration in the development of FP promotion and intervention programs.

## Data Availability

The data can be obtained by approved request from the principal investigator of the 2016 PMA2020 Indonesia survey which is The Center for Reproductive Health, Gajah Mada University, Indonesia. Address: Jl. Mahoni Block C 24, Bulaksumur, Yogyakarta 55281. Ph: + 62 274 292 4789. Email: pskespro.fkkmk@ugm.ac.id Website: https://pkr.fk.ugm.ac.id/

## Abbreviations

FP: Family Planning
SDP: Service Delivery Point
PMA: Performance Monitoring and Accountability
EA: Enumeration Area
GPS: Global Positioning System
GIS: Geographic Information System
CHW: Community Health Worker
IUD: Intra Uterine Device
CATPCA: Categorical Principal Component Analysis
AOR: Adjusted Odds Ratio
CI: Confidence Interval
PSU: Primary Sampling Unit
SE: Standard Error
AIC: Akaike information criterion
BIC: Bayesian information criterion
VIF: Variance Inflation Factors
